# Mitochondria Biogenesis and Bioenergetics Gene Profiles in Isogenic Prostate Cells with Different Malignant Phenotypes

**DOI:** 10.1155/2016/1785201

**Published:** 2016-07-10

**Authors:** Tanya C. Burch, Johng S. Rhim, Julius O. Nyalwidhe

**Affiliations:** ^1^Department of Microbiology and Molecular Cell Biology, Eastern Virginia Medical School, 651 Colley Avenue, Lester Hall Suite 424, Norfolk, VA 23507, USA; ^2^Leroy T. Canoles Jr. Cancer Research Center, Eastern Virginia Medical School, 651 Colley Avenue, Lester Hall Suite 424, Norfolk, VA 23507, USA; ^3^Department of Surgery, Uniformed Services University of the Health Sciences, 4301 Jones Bridge Road, Bethesda, MD 20814, USA

## Abstract

*Background.* The most significant hallmarks of cancer are directly or indirectly linked to deregulated mitochondria. In this study, we sought to profile mitochondria associated genes in isogenic prostate cell lines with different tumorigenic phenotypes from the same patient.* Results.* Two isogenic human prostate cell lines RC77N/E (nonmalignant cells) and RC77T/E (malignant cells) were profiled for expression of mitochondrial biogenesis and energy metabolism genes by qRT-PCR using the Human Mitochondria and the Mitochondrial Energy Metabolism RT^2^ PCR arrays. Forty-seven genes were differentially regulated between the two cell lines. The interaction and regulatory networks of these genes were generated by Ingenuity Pathway Analysis.* UCP2* was the most significantly upregulated gene in primary adenocarcinoma cells in the current study. The overexpression of* UCP2* upon malignant transformation was further validated using human prostatectomy clinical specimens.* Conclusions.* This study demonstrates the overexpression of multiple genes that are involved in mitochondria biogenesis, bioenergetics, and modulation of apoptosis. These genes may play a role in malignant transformation and disease progression. The upregulation of some of these genes in clinical samples indicates that some of the differentially transcribed genes could be the potential targets for therapeutic interventions.

## 1. Introduction

In the United States and Western Europe, prostate cancer is the most common cancer diagnosed in men and the second most common cause of cancer related deaths among men. In 2016, there will be an estimated 220,800 new cases and 27,540 deaths from prostate cancer [1]. Prostate cancer has a long latent period of development. The disease has a very heterogeneous spectrum of clinical outcomes with phenotypes ranging from indolent asymptomatic cases to very aggressive, metastatic, and lethal forms. Approximately 90% of all prostate cancers are low-grade tumors that do not metastasize. One of the most significant challenges in the management of prostate cancer is distinguishing patients with indolent asymptomatic versus the lethal forms of the disease. Currently, it is not possible to distinguish between the two forms of the disease. Many new prostate cancer biomarkers have recently emerged, but only a few have shown significant clinical value [2, 3]. Therefore, there is an urgent need to identify molecules and molecular pathways associated with the initiation and progression of prostate cancer for better diagnosis, prognosis, treatment, and management of the disease. Potential biomarkers for initiation, malignant transformation, and progression of prostate cancer, which range from the precursor lesion to organ confined primary tumor and finally to distant metastasis, may include genes, proteins, and metabolites.

Mitochondria not only are the main energy generator organelles of cells but also mediate several critical biochemical processes such as apoptosis, proliferation, and redox homeostasis. Some of the most significant hallmarks of cancer, including disabled apoptosis, invasion/metastasis, and oxidative stress, are directly or indirectly linked to deregulated mitochondria [4–10]. Therefore, the study of the expression profiles of mitochondria associated genes in isogenic cancer cells derived from the same patient but with different tumorigenic phenotypes will provide insights into molecular, biochemical, and metabolic processes that play a role in initiation, malignant transformation, and progression.

In this study, we have characterized the transcriptional profiles of mitochondria associated genes in normal and malignant isogenic human prostate cell lines derived from an African American patient by PCR array and qRT-PCR. We have used 2 different arrays to detect the expression of 84 genes involved in mitochondria-related biogenesis processes and functions and the expression of 84 genes involved in mitochondria-related bioenergetics. Although the mitochondria have noneukaryotic origins as a result of secondary endosymbiosis and possess their own chromosome, the majority of proteins that are essential for mitochondrial biogenesis and function are encoded by nuclear genomic DNA. These PCR arrays also profile nuclear encoded genes for proteins that are targeted, trafficked, and translocated into the outer and/or inner mitochondrial membranes and/or into the mitochondrial matrix. The utilization of the two arrays allows for a comprehensive evaluation of the expression profile of genes that are involved in all aspects of mitochondria biogenesis, bioenergetics, and function.

The molecular, biological, and functional categories of the differentially transcribed genes were determined by gene ontology analysis. The interaction and regulatory networks of the genes were generated and predicted by Ingenuity Pathway Analysis. Furthermore, some of the differently transcribed genes were validated in prostatectomy clinical specimens by qRT-PCR and Western blot. Several of the differentially transcribed genes may be novel markers for malignant transformation and potential drug targets for prostate cancer disease management.

## 2. Materials and Methods

### 2.1. Cell Lines, Reagents, and Tissue Samples

The two prostate cancer cell lines RC77N/E and RC77T/E that have been used in the study were established by HPV-16E6E7 immortalization of cells derived from nonmalignant and malignant tumors from radical prostatectomy specimen obtained from a 63-year-old African American prostate cancer patient [11]. This patient had clinical stage T3c adenocarcinoma with poor differentiation (Gleason 7). These cells exhibit epithelial morphology and are androgen sensitive. The RC-77T/E cells produce tumors in SCID mice whereas the RC-77N/E cells do not [11]. These cells express androgen-regulated prostate-specific homeobox gene, NKX 3.1, epithelial cell specific cytokeratin 8, androgen receptor (AR), prostate-specific antigen (PSA), and p16. Chromosome analysis showed that both cell lines are similar, near diploid human male (XY) with most chromosome counts in the 45–48 range [11]. Culture medium (Gibco Keratinocyte-serum-free medium supplemented with epidermal growth factor and bovine pituitary extract) was purchased from Invitrogen. The cells were cultured in a 5% CO_2_ humidified atmosphere, in culture medium supplemented with 50 *μ*g/mL gentamycin. The Human Mitochondria and Mitochondrial Energy Metabolism RT^2^ profiler PCR arrays were from Qiagen. The Human Mitochondria RT^2^ PCR array profiles the expression of 84 genes involved in the biogenesis and function of the mitochondria, and the Mitochondrial Energy Metabolism array profiles the expression of 84 key genes involved in mitochondrial respiration. Goat polyclonal antibodies against human UCP2 (sc-6526), rabbit polyclonal antibodies against SOD2 (sc-30080), and rabbit polyclonal antibodies against GAPDH (sc-25778) were from Santa Cruz Biotechnology Inc. All prostatectomy tissue samples were collected from patients after informed consent following Institutional Review Board-approved protocols at the Eastern Virginia Medical School. The clinical characteristics of the patients are summarized in Table 4. This study was approved by the Institutional Biosafety Committee and the Institutional Review Board at the Eastern Virginia Medical School.

### 2.2. RNA Isolation

Total RNA was extracted from prostate cells using standard procedures. Briefly, 80% confluent cells were washed multiple times with PBS and lysed directly in the culture flask with the 1 mL TRIzol reagent (Life Technologies). The resultant cell lysates were homogenized by multiple passages through a pipette. The homogenate was incubated for 5 minutes at room temperature to allow for the complete dissociation of nucleoprotein complexes. 200 *μ*L of chloroform was added to the lysate and the mixture was vigorously shaken before further incubation for 5 minutes at room temperature. The sample was centrifuged at 12,000 ×g for 15 min at 4°C and the upper aqueous phase containing RNA was carefully transferred into a new tube. The RNA was precipitated by the addition of 500 *μ*L of 100% isopropanol and incubating at room temperature for 30 minutes. The RNA was pelleted by centrifugation at 12,000 ×g for 10 minutes at 4°C. The pellet was washed with 75% ethanol and allowed to air-dry for 10 min before dissolving in RNase-free water. Dissolution efficiency was enhanced by incubating the mixture at 55°C for 15 minutes. The concentration and purity of the isolated RNA was determined by spectrophotometry using a NanoDrop® ND-1000 and by electrophoretic separation through a denaturing agarose gel.

### 2.3. First-Strand cDNA Synthesis

For cDNA synthesis, 0.5 *μ*g of total RNA was mixed with 1 *μ*L of 500 ng oligo(dT)18, 1 *μ*L of 10 mM dNTP mix, and the volume adjusted to 13 *μ*L with sterile RNase-free water. The mixture was heated to 65°C for 5 minutes to denature the RNA and incubated on ice for 1 minute. The reaction tube was centrifuged briefly to collect residual liquid from the sides of the tubes before adding 4 *μ*L 5x first-strand buffer, 1 *μ*L 0.1 M DTT, 1 *μ*L RNase inhibitor, and 1 *μ*L SuperScript III reverse transcriptase. The sample was mixed by pipetting gently and incubating at 42°C for 60 min. The reverse transcriptase was inactivated by heating at 95°C for 15 min. The reaction mixture was diluted to a final volume of 102 *μ*L with double-distilled water and stored at −80°C until being ready for use.

### 2.4. Real-Time PCR Array for Mitochondria-Related Gene Expression

The Human Mitochondria (PAHS-087Z) and Mitochondrial Energy Metabolism (PAHS-008YA) RT2 profiler PCR arrays (Qiagen) were used to determine the expression profiles of genes that are related to mitochondrial biogenesis and bioenergetics functions by qRT-PCR. The Human Mitochondria RT^2^ PCR array profiles the expression of 84 genes involved in the biogenesis and function of the mitochondria; this includes genes that are involved in membrane polarization and potential, small molecule transport, targeting proteins to mitochondria, mitochondrion protein import, outer membrane translocation, inner membrane translocation, mitochondrial fission and fusion, mitochondrial localization, and apoptosis. The Mitochondrial Energy Metabolism array profiles the expression of 84 key genes involved in mitochondrial respiration; this includes complex I (NADH-coenzyme Q reductase) genes, complex II (succinate-coenzyme Q reductase) genes, complex III (coenzyme Q-cytochrome c reductase) genes, complex IV (cytochrome c oxidase) genes, complex V (ATP Synthase) genes, and some pathway activity signature genes. For the qRT-PCR analysis, a reaction mixture comprising of 550 *μ*L of 2x RT^2^ SYBR® Green qPCR Mastermix, 102 *μ*L of the diluted first-strand cDNA synthesis reaction, and 448 *μ*L of ddH2O was prepared. The cocktail was then added to the PCR array. Real-time PCR detection was performed on a Bio-Rad® iCycler under the following conditions: 95°C for 10 min, 40 cycles of 95°C for 15 sec, and 60°C for 1 min. The results were analyzed by the ΔΔCt method.

### 2.5. Statistical Analyses

The RT-PCR data were analyzed using the ΔΔCt module at the Qiagen Gene Globe Data Analysis Center portal: http://www.qiagen.com/us/shop/genes-and-pathways/data-analysis-center-overview-page/. The real-time PCR module transforms threshold cycle (Ct) values to calculate results for gene expression. The efficiency of all the primers used in the kits has been shown to be over 90%. The RT-PCR arrays contain control wells/samples for the determination and/or verification of human genomic contamination, reverse transcription control, and positive PCR controls. At least five reference genes including beta-actin (ACTB), glyceraldehyde-3-phosphate dehydrogenase (GAPDH), beta-2-microglobulin (B2M), hypoxanthine phosphoribosyltransferase 1 (HPRT1), and ribosomal protein, large, P0 (RPLP0) were used for data normalization. For these analyses, genes with fold change >2 in expression at a *p* value of < 0.05 between RC77T/E and RC77N/E were defined as differentially expressed and selected for inclusion in comparative analyses of the association of gene expression and malignant transformation.

### 2.6. Western Blot Validation of Differential Expressed Genes

Western blot was used to confirm the expression profiles of proteins encoded by mitochondria-related genes using normalized concentration of cell lysate samples as we have previously described [12]. Briefly, confluent cells were harvested and total proteins were extracted after the lysis by incubation in RIPA buffer cell buffer (10 mM Tris-HCl, pH 7.4, 100 mM NaCl, 1 mM EDTA, 1 mM EGTA, 1% Triton, 0.1% SDS, and 10% glycerol with protease inhibitors) at 4°C for 30 minutes with vortexing at 10 minutes' intervals. After centrifugation at 13,000 rpm for 10 minutes at 4°C, the supernatants were obtained and transferred to fresh tubes. Protein concentrations were determined using BCA protein assay (Thermo Scientific, Rockford, IL). Normalized protein concentrations (40 *μ*g) from the cell lines were separated by SDS-PAGE and transferred to PDVF membranes (Millipore, Billerica, MA) using standard methods. Membranes were blocked in LiCor*™* blocking buffer (Rockland Immunochemicals, Gilbertsville, PA, USA). The membranes were incubated with the respective primary antibodies at a concentration of 0.2 *μ*g/mL overnight at 4°C. The membranes were washed extensively with 0.1% Tween-20 in PBS before incubation with species-specific IRDye700 or 800-conjugated secondary antibodies (goat anti-rabbit, 1 : 20,000; donkey anti-mouse, 1 : 20,000; and donkey anti-goat, 1 : 20,000) for 1 h at room temperature. Target protein bands were visualized using a LiCor Odyssey Infrared Imager (LiCor, Lincoln, NE).

### 2.7. Glucose Uptake and Lactate Secretion Assays

The consumption of glucose and secretion of lactate in the medium during cell culture over a 24 h period were compared using identical numbers of the RC77N/E and RC77T/E cells in a bioluminescent assay that couples glucose and lactate oxidation and NADH production with NADH detection using a reductase/luciferase detection system. The relative luminescence signals corresponding to the glucose and lactate that was present in the medium after the incubation period were determined from triplicate experiments that included controls for normalization. The luminescence signals were measured using a Glomax 96 Microplate Luminometer (Promega).

## 3. Results and Discussion

### 3.1. Comparative Mitochondria Associated Gene Expression Profiles between RC77N/E and RC77T/E Cell Lines

**Figure 1:**
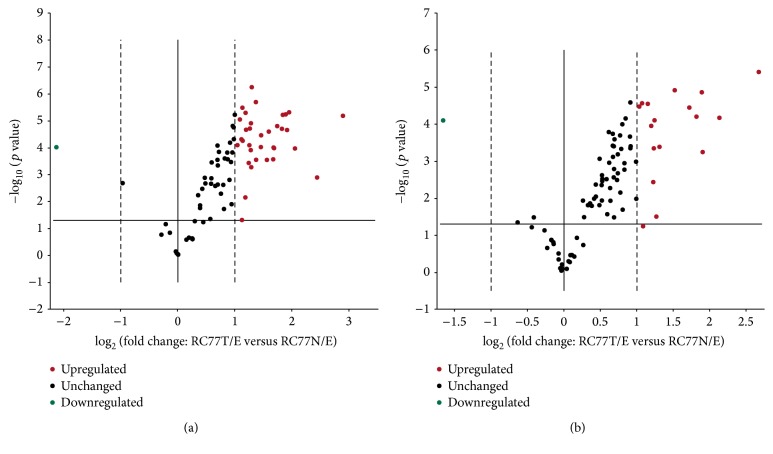
Volcano plots of the mitochondria real-time PCR array results. The continuous vertical line indicates a 1.0-fold change in gene expression. The dotted vertical lines indicate the desired threshold of a 2.0-fold change in gene expression. The horizontal continuous line indicates the desired 0.05 threshold for the *p* value of the *t*-test. (a) represents the Human Mitochondria array and (b) represents the mitochondria bioenergetics array.

In the current study, we have determined and validated the transcriptional profiles of genes that are associated with mitochondria biogenesis and bioenergetics using isogenic prostate cell lines derived from the same patient. The two cell lines are cytogenetically similar but with different tumorigenic phenotypes: RC77N/E is nonmalignant whereas RC77T/E is malignant. The RC-77T/E cell line derived from malignant tumor was able to form larger three-dimensional spheroids in RWV system and form tumors in SCID mice compared to RC-77N/E cell line [11]. The expression profiles of genes that are related to mitochondria biogenesis, function, metabolism, and bioenergetics were compared using quantitative real-time PCR microarray technology. The concentration of extracted RNA was determined by spectrophotometry and the purity confirmed by electrophoretic resolution through a denaturing agarose gel prior to RT-PCR. A total of 168 genes related to mitochondrial function were compared between the two cell lines (malignant versus nonmalignant). Forty-seven of the 168 genes were significantly up/downregulated (fold change >2.0, *p* < 0.01) in the malignant RC77T/E cells compared to RC77N/E cells (Figure 1). The data is summarized in Table 1. Forty-six genes were significantly upregulated (fold change > 2.0, *p* < 0.01) in RC77T/E versus RC77N/E and one gene (NEFL) is significantly downregulated in RC77T/E versus RC77N/E (fold change < 2.0, *p* < 0.01). The genes, which have significant differential up/downregulation, have biochemical, molecular, and physiological functions in the mitochondria. These include oxidative phosphorylation (OXPHOS), apoptosis, membrane polarization and potential, small molecule transport, mitochondrial transport, outer and inner membrane translocation, targeting proteins to the mitochondria, mitochondrial protein import, mitochondrial fission and fusion, and mitochondrial localization.

### 3.2. Expression Profiles of Oxidative Phosphorylation Associated Genes

The crucial role of mitochondria in carcinogenesis is partly because they are the main source of endogenous reactive oxygen species (ROS) that escape from the electron transport chain during oxidative phosphorylation process. The effect of ROS in carcinogenesis is multifaceted and may act as a double-edged sword. First, ROS make both nuclear DNA and mitochondrial DNA susceptible to damage, and mutations in these two DNA pools are reported to lead to carcinogenesis [6]. Second, ROS are crucial intermediates of cellular signaling that promote and suppress tumorigenesis simultaneously [6], and changes to cancer cell mitochondria metabolism may modulate tumor formation and disease progression [7]. Our study demonstrates that there is a general increase in expression of the genes that are involved in oxidative phosphorylation in RC77T/E compared to RC77N/E. There are 33 complex I genes represented in the arrays; 6 genes including* NDUFA8*,* NDUFB6*,* NDUFC1*,* NDUFS5*,* NDUFS7,* and* NDUFV2* are significantly upregulated in the malignant RC77T/E compared to RC77N/E. The most significantly upregulated complex I gene is* NDUFC1* with a 4.3791-fold increase. The respiratory complex II is the only mitochondrial enzyme complex that participates in both the citric acid cycle and the electron transport chain. The expression profiles of the four complex II genes in the array,* SDHA*,* SDHB*,* SDHC,* and* SDHD,* are similar in the two cell lines. Only one of the six respiratory complex III genes in the arrays,* UQCRQ*, is upregulated in RC77T/E when compared to RC77N/E cells. Cytochrome c oxidase or complex IV is the last enzyme in the respiratory electron transport chain of mitochondria. The mitochondria metabolism and bioenergetics arrays that we used contain 13 complex IV genes. The expression profiles of complex IV genes in the two cell lines are similar. Mitochondria complex V comprises a class of enzymes that catalyze the decomposition of ATP into ADP and a free phosphate group (PO_4_^3−^). The dephosphorylation reaction releases energy that is used for cellular processes. Thirteen genes belonging to complex V are represented in the array and 5 genes* ATP5F1*,* ATP5G1*,* ATP5G3*, and* ATP5I* are significantly upregulated in the malignant RC77T/E cells compared to the nonmalignant RC77N/E cells. The most significantly upregulated complex V gene is ATP5L with a 3.7348-fold increase in the malignant RC77T/E cells.

To counteract the detrimental effects of ROS that includes nuclear DNA and mitochondrial DNA damage, all living cells encode genes for superoxide dismutases, enzymes that catalyze the dismutation of the superoxide (O_2_^−^) radical into either ordinary molecular oxygen or hydrogen peroxide. The hydrogen peroxide which may still be damaging to cells is oxidized to water and oxygen by catalase. The three most common superoxide dismutases genes are SOD1, SOD2, and SOD3. The three genes are encoded by the nuclear genome but are localized in different cellular compartments. SOD1 is cytoplasmic; SOD2 is trafficked to the mitochondria, while SOD3 is extracellular. SOD1 and SOD2 are included in the mitochondrial biogenesis array and both of them are significantly upregulated in the malignant RC77T/E cells compared to the nonmalignant RC77N/E cells. The upregulation of these genes may be in response to the generation of higher amounts of ROS by the malignant RC77T/E cells that would necessitate the expression and translation of SOD1 and SOD2 to detoxify the reactive oxygen species. In addition to oxidative phosphorylation, mitochondria are also involved in the oxidation of lipids. This process is modulated by two inner mitochondrial membrane proteins that are encoded by two nuclear genes CPT1 and CPT2. These proteins oxidize long-chain fatty acids in the mitochondria. CPT1 is preferentially expressed in muscles. In our current analysis, CPT2 is significantly upregulated in the malignant RC77T/E cells compared to the nonmalignant RC77N/E cells, which suggests there is an increase in the oxidation of long-chain fatty acids in the malignant cells.

### 3.3. Expression Profiles of Apoptosis Modulating Genes

One of the other crucial roles of mitochondria in carcinogenesis is the control and mediation of apoptosis. BAK1 (BCL2-antagonist/killer 1) and BID (BH3 interacting domain death agonist) are promoters of apoptosis whereas BCL2-like 1 inhibits apoptosis. The expression of BAK1, BCL2L1, and BID is significantly upregulated in the malignant RC77T/E cells. It is possible that BAK and BID are regulated further at the posttranscriptional level by miRNA as has been shown for mir125b [13, 14].

The expression of the cyclin-dependent kinase inhibitor 2A (CDKN2A) gene, a negative regulator of proliferation of normal cells, also shows a significant fourfold increase in the malignant RC77T/E versus the nonmalignant RC77N/E cells. There is also a significant twofold upregulation in the expression of* TP53* gene. TP53 has multiple roles in malignant transformation and carcinogenesis. These include apoptosis, genomic stability, and inhibition of angiogenesis. MIRO1 and MIRO2 (mitochondrial Ras homolog gene family, members T1 and T2), also referred to as* RHOT1* and* RHOT2*, belong to the mitochondrial Rho GTPase family. These genes encode for the proteins RHOT1 and RHOT2, which are involved in mitochondria homeostasis and apoptosis. In the present study, although there are no differences in the expression levels of* RHOT1*,* RHOT2* is 2-fold upregulated in the malignant RC77T/E versus the normal RC77N/E cells.

### 3.4. Expression Profiles of Molecular Chaperones and Molecular Transporter

Multiple heat shock proteins/chaperones are also significantly upregulated in the malignant RC77T/E cells. These include the genes for the mitochondria resident chaperones* HSPD1* and* GRPEL1* and also cytosolic molecular chaperones* HSPA1A*,* HSPA1B,* and* HSP90AA1*. The other genes that are upregulated in the malignant RC77T/E cells are mostly involved in small molecule transport, mitochondrial transport, membrane polarization and potential, mitochondria fission, and mitochondria protein import. The small molecule transporters that are significantly upregulated include* SLC25A13*,* SLC25A19*,* SLC25A20*,* SLC25A22*,* SLC25A25*,* SLC25A3*, and* SLC25A37*.* DNM1L*, which encodes for Dynamin-1-like protein, a GTPase that regulates mitochondrial fission, was also over 3-fold significantly upregulated in RC77T/E compared to RC77N/E cells. A role for Drp1 in cell cycle progression, genome instability, cell migration, and apoptosis in cancer cells has also been recently uncovered. Another significantly upregulated gene was* MSTO1*, a gene encoding for the mitochondrial distribution and morphology regulator protein. Human Misato has a role(s) in mitochondrial distribution and morphology and its unregulated expression leads to cell death. Ten mitochondria protein import translocase genes for the inner and the outer mitochondria,* TIMM10B*,* TIMM17A*,* TIMM17B*,* TIMM22*,* TIMM23*,* TIMM50*,* TIMM8B*,* TIMM34*,* TIMM40*, and* TIMM40L,* are also significantly upregulated in RC77T/E compared to RC77N/E.

### 3.5. NEFL Is the Most Significantly Downregulated Gene between RC77T/E and RC77N/E Cell Lines

The neurofilament light polypeptide (*NEFL*) gene located on chromosome 8q21 is associated with the cancer of several organs and is regarded as a potential tumor suppressor gene. The gene encoded by the protein is also associated with mitochondria localization. The* NEFL* gene was the most significantly downregulated gene in RC77T/E compared to RC77N/E cells. The low expression levels of* NEFL* may be directly correlated with the malignant transformation that is observed in RC77T/E. Recent studies suggest that loss of heterozygosity at the gene locus and DNA methylation-mediated silencing of the neurofilament genes* NEFH*,* NEFM*, and* NEFL* are frequent events that may contribute to the progression of breast cancer and possibly other malignancies [15, 16]. The RT-PCR showing the ΔΔCt values for NEFL in the two cell lines is shown in Supplemental Figure  1 (in Supplementary Material available online at http://dx.doi.org/10.1155/2016/1785201), which clearly demonstrates the downregulation of the gene in the malignant RC77T/E cells compared to the nonmalignant RC77N/E. Our observations are supported by the data from The Cancer Genome Atlas where the copy numbers of the gene are significantly lower in a majority of the cases from both acinar prostate adenocarcinoma and prostate adenocarcinoma cases (Supplemental Figure  2).

### 3.6. UCP2 Is the Most Significantly Upregulated Gene between RC77T/E and RC77N/E Cell Lines

**Figure 2:**
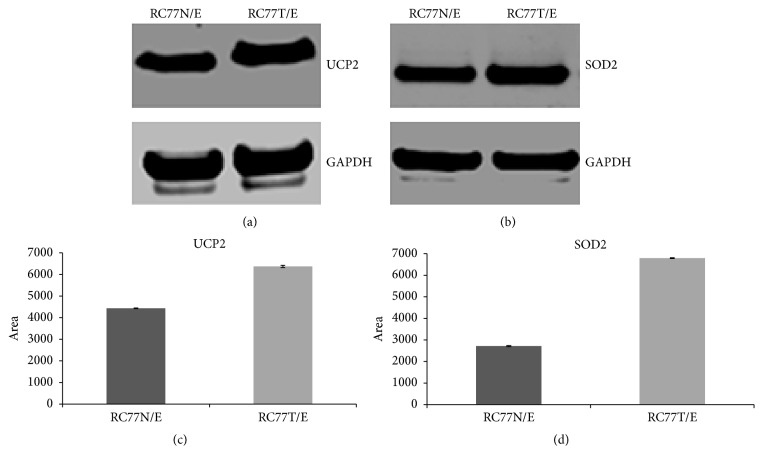
Validation of differential expression of mitochondrial uncoupling protein-2 and SOD2 in RC77N/E and RC77T/E in cell lysates. Normalized total protein lysates from the two cell lines were subjected to Western blot analysis to detect UCP2 (a) and SOD2 (b). All the samples were normalized with respect to GAPDH, which was included as a loading control. The corresponding ImageJ and normalized densitometry comparisons are shown in (c) and (d), respectively.

**Figure 3:**
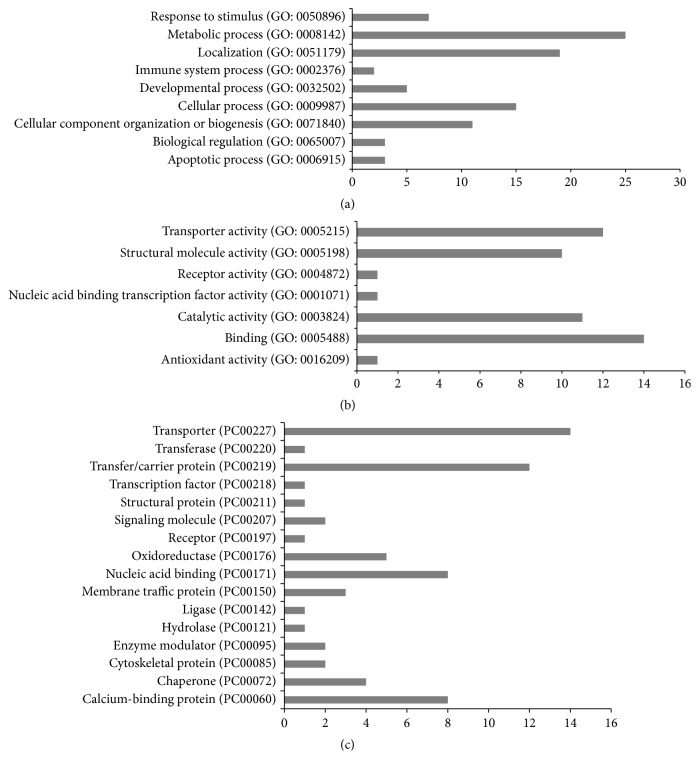
GO analysis of differentially transcribed genes. The differentially transcribed genes were described in 3 categories: (a) biological process, (b) molecular function, and (c) protein classes. GO terms were used to describe the attributes of the differentially transcribed genes.

In our current RT-PCR analysis comparing isogenic RC77T/E and RC77N/E cells,* UCP2*, which is a member of the family of uncoupling proteins located in the inner mitochondrial membrane, was the most significantly upregulated gene with a 10-fold upregulation in the malignant cells.* UCP2* uncouples electron transport from ATP production. Importantly, the expression profiles of the genes of the two other uncoupling proteins that are represented,* UCP1* and* UCP3*, in the array are identical. UCP2 function has been previously linked to obesity and diabetes. The role of UCP2 in cancers is not well understood; however, recently, the tumor promoting properties of UCP2 were described in vitro and in vivo in a mouse xenograft model. Further biochemical analysis of the protein expression in the two cell lines reveals an upregulation of the protein levels in RC77T/E compared to RC77N/E cells albeit with a lower fold upregulation (Figure 2(a)). This would suggest that both transcriptional and posttranscriptional mechanisms may be involved in regulating the role of* UCP2* in malignant transformation. The observed lower levels in the fold difference of the protein could also be attributed to the longevity of the protein in the cells. UCP2 protein is reported to be highly unstable, with half-life of 30 min and a rapid turnover by the cytosolic proteasome [17]. The upregulation of SOD2 at protein level in the malignant RC77T/E cells was also confirmed by Western blot (Figure 3(b)). Densitometry analysis and *t*-tests were to confirm the differential expression of the proteins in the cell lines (Figures 2(c) and 2(d)).

### 3.7. Gene Ontology Analysis of Differentially Regulated Genes

Gene ontology terms were used to describe three attributes of the differentially transcribed mitochondria associated proteins: biological process, molecular function, and protein class, using DAVID and Panther Databases [18–20]. The differentially transcribed genes were closely related to biological process such as protein apoptosis, cellular component organization or biogenesis, and metabolic processes. In addition, they were associated with molecular functions like antioxidant activity, binding activities, catalytic activities, and transporter activity (Figures 3(a)–3(c)).

### 3.8. Ingenuity Pathway Analysis of Differentially Regulated Genes in RC77T/E versus RC77N/E Cell Lines

The interpretation of high-throughput gene expression data is greatly facilitated by the consideration of prior biological knowledge, which facilitates the meaningful interpretation of expression data. Networks constructed from individual relationships curated from the literature are particularly suited for this task, since they create mechanistic hypotheses that explain the expression changes observed in datasets. The changes in molecular processes, molecular functions, and genetic networks in RC77N/E and RC77T/E cells were further evaluated by analyzing differentially regulated genes using ingenuity pathways analysis (IPA) (Ingenuity Systems®, http://www.ingenuity.com/). IPA software application enables the identification of the biological mechanisms, pathways, and functions most relevant to their experimental datasets or genes of interest (Qiagen). The top canonical pathways and their respective probability scores are summarized in Table 2. The pathways with the most significant probability scores (significance threshold 2.0, −log⁡(*p*  value)) are related to mitochondrial dysfunction, oxidative phosphorylation, induction of apoptosis, and amyotrophic lateral sclerosis (ALS) signaling. The cause of ALS is currently unknown but appears to involve different mechanisms including oxidative damage.

### 3.9. Regulatory Network Analysis by IPA

All of the forty-seven differentially transcribed mitochondria associated genes were uploaded to the database to analyze upstream regulatory events. The identified molecules, annotations, *p* value of overlap, and target molecules of the upstream regulators in the regulatory network are described and listed in Table 3 and Supplemental Figure  3. The significance threshold for the *p* values was set at <0.05. The upstream regulator with the lowest *p* value at 4.25*E* − 08 is RBM5 also known as RNA-binding protein 5, RNA-binding motif protein 5, or putative tumor suppressor LUCA15. The targets of interest among the genes that have been analyzed include* ATP5G3*,* CDKN2A*,* HSP90AA1*,* NDUFV2*, and* UCP2*. RBM5 is a component of the spliceosome A complex and it regulates alternative splicing of a number of mRNAs. RBM5 may both positively and negatively regulate apoptosis by regulating the alternative splicing of several genes involved in this process, including FAS and CASP2/caspase-2 [21–27]. In our analyses, RBM5 is predicted to be activated in the malignant RC77T/E cells and it upregulates the expression of ATP5G3 while inhibiting and downregulating the expression of* CDKN2A*,* HSP90AA1*,* NDUFV2*, and* UCP2*. The upstream regulator with the second lowest *p* value at 1.43*E* − 05 is AIFM1, mitochondrial apoptosis-inducing factor 1, also known as programmed cell death protein 8. AIFM1 regulates mitochondria complex I activities and also has a role in apoptosis [28–30]. Our data predicts that AIFM1 is activated in the malignant cells and it upregulates the expression of the two complex I genes* NDUFB6* and* NDUFS7*. Beta defensin is also significantly upregulated in malignant cells in the IPA upstream analysis, and it is predicted to activate and upregulate the expression of* BCL2L1*. It also inhibits the expression of* BID* with associated downregulation of the genes. This is consistent with the antiapoptotic and proapoptotic functions of the two genes, respectively.

The IPA data shows that transcription regulators control multiple mitochondria associated genes. For example, the heat shock transcription factor 1 (HSF1) acts to regulate the transcription of four different genes, namely, BCL2L1,* HSP90AA1*, and* HSPA1A/HSPA1B*. HSF1 is a DNA-binding protein that specifically binds heat shock promoter elements (HSE) and activates transcription [31].* BCL2L1*,* HSP90AA1*, and* HSPA1A/HSPA1B* genes are differentially regulated by HSF1 in the malignant RC77T/E cells. The IPA data predicts the activation of BCL2L1 gene transcription and the inhibition of transcription of* HSP90AA1* and* HSPA1A/HSPA1B*. Another transcription factor BGT2, also known as NGF-inducible antiproliferative protein PC3, activates and upregulates the transcription of* SOD1* and* SOD2*, which are also activated and upregulated by NFE2L2 (nuclear factor erythroid 2-related factor 2) in the malignant cells. NFE2L2 is a transcription activator that binds to antioxidant response elements (ARE) in the promoter regions of target genes, and it is important for the coordinated upregulation of genes in response to oxidative stress [32].

Transcription factor RelB is also predicted to activate and upregulate the transcription of both* BCL2L1* and* SOD2*. RelB is a component of the NF-kappa-B complex [33]. Our results show that the different transcription regulators have different degrees of regulation on the expression of the mitochondria associated genes and these influences could be measured by the *p* value of overlap. The smaller the *p* value of overlap is, the greater the influence of the regulation is expected. The consistent predictions of independent transcription factors having identical effects/regulation of specific genes/proteins would imply that specific pathways activate multiple pathways in malignant cells compared to the normal cells. Based on these observations, it can be concluded that multiple transcription factors modulate the expression of genes that control mitochondrial function. This results in the upregulation of specific genes, for example,* UCP2*,* SOD2*,* SOD1*,* CDKN2A*,* BCL2L1*,* HSP90AA1*,* HSPA1A*,* SLC25A19,* and* BAK1,* and downregulation of NEFL, respectively. This is also associated with the activation of specific pathways, for example, TP53 pathway. Based on the PCR arrays that we have used and the *p* values of overlap of the upstream regulators, the transcription factors that are possibly involved in malignant transformation and changes in the phenotypes that are observed between the two cell lines would be in the following order, BGT2 > NFE2L2 > RelB > HSF1. It is important to note that SOD2 is a central target of multiple upstream regulators. Aside from regulators, current data suggests that specific miRNA is associated with the expression of some of the genes. mir-491 is predicted to regulate* BCL2L1* in the current analysis. The mature products of mir-491 (miR-491-5p and miR-491-3p) have been reported to possess different functions in different cancers [34–36].

Out of the 149 statistically significant upstream regulators (*p* < 0.05), 31 have* SOD2* as a predicted target which is the second highest one after* CDKN2A* (Cyclin-Dependent Kinase Inhibitor 2A) at 45. CDKN2A is involved in numerous processes that control cell cycle progression, cell proliferation, and apoptosis. This data supports the hypothesis that SOD2 plays a critical role in malignant transformation and disease progression in prostate and other cancer cells. Although there are contradictory reports about the role of SOD2 in tumorigenesis, the overexpression of SOD2 in the current study is consistent with previous studies [37, 38]. These results highlight and support the hypothesis that redox imbalance is one of the key features that occur during malignant transformation, and this may favor migration and invasion, leading to a more aggressive cancer phenotype. SOD2 overexpression may enhance malignant transformation, invasiveness, and migration by signaling events that drive metastasis or by attenuating catalase activity [39, 40].

### 3.10. Comparative Glucose Uptake and Lactate Secretion by RC77N-E and RC77T-E Cell Lines

**Figure 4:**
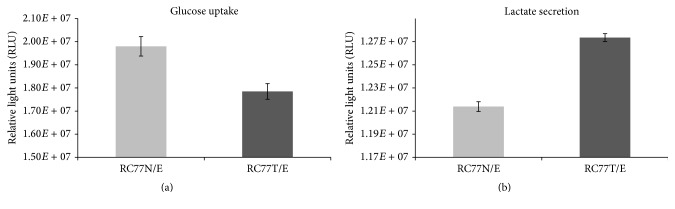
Glucose uptake and lactate secretion in RC77N/E and RC77T/E cells. Glucose consumption (a) and lactate secretion (b) in culture medium were determined after growing RC77N/E and RC77T/E for 24 hours. The data is from triplicate experiments and there is a significant difference (*p* < 0.05) in the uptake of glucose and secretion of lactate by the two cell lines.

Many tumor cells exhibit rapid glucose consumption, with most of the glucose-derived carbon secreted as lactate despite abundant oxygen availability (the Warburg effect). We compared the glucose uptake and lactate secretion by the nonmalignant RC77N-E and the malignant RC77T-E cells to determine if there were differences in their glucose metabolism process. The results show that there is a significant difference in the glucose uptake and lactate secretion by the two cell lines. The malignant RC77T-E cells consume glucose from the medium more rapidly than their nonmalignant counterparts (Figure 4(a)) and they also secrete significantly higher amounts of lactate (Figure 4(b)). Based on these observations, it can be concluded that the RC77T-E cells exhibit the Warburg effect and the gene expression profiles that are observed are potentially related to the changes in metabolism and metabolic pathways between the two cells [41, 42]. Citrate which is a major substrate for generation of energy in most cells is produced in mitochondria and either used in the Krebs cycle or released into cytoplasm through a specific mitochondrial carriers. It has been suggested that, due to a metabolic switch, neoplastic cells would become citrate-oxidizing, unlike normal prostatic cells that show a low citrate-oxidizing capability [43, 44]. The mitochondrial citrate transporter (SLC25A1, tricarboxylate transport protein) maintains mitochondrial homeostasis in metabolically active, high proliferating tissues including cancer cells. In our current study, the SLC25A1 is not significantly differentially expressed between the two cell lines using a significant threshold cut-off of 2.0 for fold increase. Based on these results, it would be possible that citrate metabolism is not significantly altered between the two cell lines.

### 3.11. Validation of Increased Expression of UCP2 in Prostate Adenocarcinoma Tissues by Western Blot

**Figure 5:**
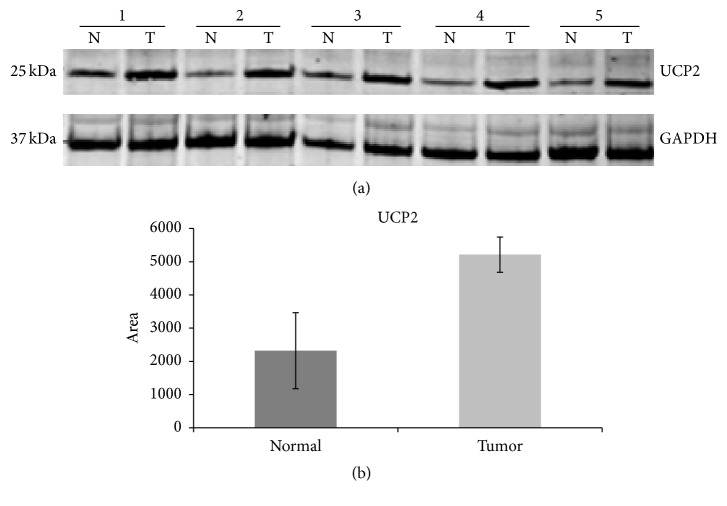
Validation of differential expression of mitochondrial uncoupling protein-2 in prostatectomy tissues (a). Normalized total protein lysates from five prostatectomy tissues, normal and tumor from the same patient, were subjected to Western blot analysis to detect UCP2. GAPDH detection was included as a loading control.

The expression level of UCP2 was found to be significantly increased in the malignant RC77T/E cells compared to the nonmalignant RC77N/E cells in our RT-PCR array analysis. The expression level of these two proteins was evaluated by Western blot analysis using protein extracts from prostatectomy tissue samples. Normalized concentrations of protein were obtained from normal adjacent tissues and tumor tissues upon pathological examination and macrodissection of prostatectomy tissues. The characteristic features of the patients are shown in Table 4. The relative abundance of UCP2 in the normal adjacent tissues was compared to the adenocarcinoma tissues by densitometry in triplicate experiments using GAPDH as control for normalization (Figure 5). The statistical *t*-test results show that UCP2 is significantly upregulated in the tumor tissues compared to the normal adjacent tissues in the five clinical samples. Our data is consistent with recently published data that demonstrates an upregulation of UCP2 in human head and neck, skin, prostate, and pancreatic tumor compared to normal adjacent tissues [45]. It has been suggested that UCP2 overexpression protects cancer cells from reactive oxygen species induced apoptosis [46]. Additionally, UCP2 may also promote and shift cancer cell metabolism from glucose oxidation to fatty acid oxidation [47–49]. The reprogrammed metabolism may not only play a role in malignant transformation and maintenance of the phenotype but also be involved in chemoresistance that is associated with tumorigenic cells. The validation in clinical prostate cancer specimens showed that there are significant differences in the expression levels of UCP2 between tumor and nontumor tissues. The evidence provided suggests that some of the differentially transcribed genes are potential novel drug targets and biological markers for disease prediction. We are currently working on validating the transcription levels of some of the other remaining differentially transcribed genes.

## 4. Conclusions

This is the first study to focus on transcriptional profiling of mitochondria biogenesis and bioenergetics genes that may play a role in initiation, malignant transformation, and disease progression in prostate cancer by PCR array using isogenic prostate cell lines derived from the same patient. Differentially transcribed genes were analyzed by GO analysis, and their regulatory networks were determined. The pathways with the most significant probability values are related to mitochondrial dysfunction, oxidative phosphorylation, and superoxide radical degradation. These genes may play a role in malignant transformation and disease progression. The upregulation of some of these genes in clinical samples indicates that some of the differentially transcribed genes could be the potential targets for therapeutic interventions and are also the potential molecular markers to predict prostate cancer malignancy transformation.

## Supplementary Material

RTPCR was performed for the 168 genes in the arrays and the results were analyzed by analyzed by the ΔΔCt method. Supplemental Figure 1 shows the ΔΔCt values for NEFL which is differentially expressed between the two cell lines. There is a significant down regulation of NEFL gene in the malignant RC77T/E cells compared to the non-malignant RC77N/E cells. The Cancer Genome Atlas (TCGA) was queried to determine NEFL gene copy numbers in human prostate tissue samples. The copy numbers of the gene are significantly lower in a majority of the cases from both acinar prostate adenocarcinoma and prostate adenocarcinoma cases (Supplemental Figure 2). Ingenuity pathway analysis was used to generate regulatory networks of the differentially transcribed genes between the two cell lines and the networks are summarized in Supplemental Figure 3.

## Figures and Tables

**Table 1:** Differentially expressed mitochondria associated genes in RC77T/E versus RC77N/E.

Gene ID	Gene	Fold change	*p* value	Mitochondrial function
*ATP5F1*	ATP synthase F(0) complex subunit B1	2.335	0.003692	Complex V (ATP synthase)
*ATP5G1*	ATP synthase F(0) complex subunit C1	3.29	0.000036	Complex V (ATP synthase)
*ATP5G3*	ATP synthase F(0) complex subunit C3	2.3634	0.000079	Complex V (ATP synthase)
*ATP5L*	ATP synthase, mitochondrial F0 complex, subunit E	3.7348	0.000572	Complex V (ATP synthase)
*BAK1*	BCL2-antagonist/killer 1	5.4275	0.001285	Apoptosis, membrane polarization and potential
*BCL2L1*	BCL2-like 1	2.5872	0.000284	Apoptosis, membrane polarization and potential
*BID*	BH3 interacting domain death agonist	2.1975	0.000056	Apoptosis, membrane polarization and potential
*CDKN2A*	Cyclin-dependent kinase inhibitor 2A	3.863	0.000005	Apoptosis
*CPT2*	Carnitine palmitoyltransferase 2	2.2704	0.007064	Mitochondrial transport, import of palmitoyl-CoA
*DNM1L*	Dynamin 1-like	3.3432	0.000016	Apoptosis, mitochondrial localization
*GRPEL1*	GrpE-like 1, mitochondrial	3.1984	0.000099	Targeting proteins to mitochondria, protein import
*HSP90AA1*	Heat shock protein 90 kDa alpha (cytosolic), class A member 1	3.7062	0.000006	Mitochondrial transport, molecular chaperone
*HSPA1A*	Heat shock 70 kDa protein 1A	2.478	0.000413	Molecular chaperone
*HSPA1B*	Heat shock 70 kDa protein 1B	2.405	0.031481	Molecular chaperone
*HSPD1*	Heat shock 60 kDa protein 1 (chaperonin)	2.1902	0.000003	Mitochondrial transport, mitochondria chaperone
*MSTO1*	Misato homolog 1	2.3655	0.000371	Mitochondrial localization, distribution and morphology
*NDUFA8*	NADH dehydrogenase [ubiquinone] 1 alpha subcomplex subunit 8	2.864	0.000012	Complex I (NADH-coenzyme Q reductase)
*NDUFB6*	NADH dehydrogenase [ubiquinone] 1 beta subcomplex subunit 6	3.705	0.000014	Complex I (NADH-coenzyme Q reductase)
*NDUFC1*	NADH dehydrogenase [ubiquinone] 1 subunit C1, mitochondrial	4.3791	0.000068	Complex I (NADH-coenzyme Q reductase)
*NDUFS5*	NADH dehydrogenase (ubiquinone) Fe-S protein 5	2.0443	0.000034	Complex I (NADH-coenzyme Q reductase)
*NDUFS7*	NADH dehydrogenase (ubiquinone) Fe-S protein 7	2.1198	0.057441	Complex I (NADH-coenzyme Q reductase)
*NDUFV2*	NADH dehydrogenase (ubiquinone) flavoprotein 2	3.5214	0.000063	Complex I (NADH-coenzyme Q reductase)
*NEFL*	Neurofilament, light polypeptide	−4.3762	0.000097	Mitochondrial localization, potential tumor suppressor
*RHOT2*	Ras homolog gene family, member T2	2.0615	0.000081	Mitochondrial localization, mitochondrial trafficking
*SLC25A13*	Solute carrier family 25, member 13 (citrin)	3.7679	0.000022	Small molecule transport, aspartate glutamate carrier
*SLC25A19*	Solute carrier family 25 (thiamine pyrophosphate carrier), member 19	2.4244	0.000124	Small molecule transport, thiamine pyrophosphate carrier
*SLC25A20*	Solute carrier family 25 (carnitine/acylcarnitine), member 20	3.5411	0.00002	Small molecule transport, transport of acylcarnitines
*SLC25A22*	Solute carrier family 25 (glutamate carrier), member 22	3.1844	0.000272	Small molecule transport, glutamate/H(+) symporter 1
*SLC25A25*	Solute carrier family 25 (phosphate carrier), member 25	3.2177	0.000105	Calcium-dependent mitochondrial solute carrier
*SLC25A25*	Solute carrier family 25 (phosphate carrier), member 25	2.3477	0.000454	Calcium-dependent mitochondrial solute carrier
*SLC25A3*	Solute carrier family 25 (phosphate carrier), member 3	2.1644	0.000049	Small molecule transport, phosphate transport
*SLC25A37*	Solute carrier family 25, member 37	2.7408	0.000096	Small molecule transport, mitochondrial iron transporter
*SOD1*	Superoxide dismutase 1	2.5797	0.000002	Membrane polarization and potential, detoxification of ROS
*SOD2*	Superoxide dismutase 2, mitochondrial	2.4298	0.000013	Apoptosis, destruction of superoxide anion radicals
*TIMM10B*	Mitochondrial import inner membrane translocase subunit	2.384	0.000082	Protein import, inner membrane translocation
*TIMM17A*	Translocase of inner mitochondrial membrane 17A	2.3979	0.00002	Inner membrane translocation
*TIMM17B*	Translocase of inner mitochondrial membrane 17B	2.1244	0.000009	Inner membrane translocation
*TIMM22*	Translocase of inner mitochondrial membrane 22	2.9497	0.000287	Inner membrane translocation
*TIMM23*	Translocase of inner mitochondrial membrane 23	2.276	0.000005	Inner membrane translocation
*TIMM50*	Translocase of inner mitochondrial membrane 50	2.4393	0.000534	Inner membrane translocation
*TIMM8B*	Translocase of inner mitochondrial membrane 8B	2.2896	0.000022	Inner membrane translocation
*TOMM22*	Translocase of outer mitochondrial membrane 22	2.4539	0.000001	Mitochondrial import receptor
*TOMM34*	Translocase of outer mitochondrial membrane 34	3.579	0.000006	Outer membrane translocation
*TOMM40*	Translocase of outer mitochondrial membrane 40	3.0187	0.000026	Outer membrane translocation
*TOMM40L*	Translocase of outer mitochondrial membrane 40-like	4.1483	0.000108	Outer membrane translocation
*TP53*	Tumor protein p53	2.0973	0.000006	Tumor suppressor
*UCP2*	Uncoupling protein 2	10.5781	0	Transport, uncoupling oxidative phosphorylation
*UQCRQ*	Cytochrome b-c1 complex subunit 8	2.2169	0.000029	Complex III (coenzyme Q-cytochrome c reductase)

**Table 2:** Top five canonical pathways associated with differentially expressed genes between RC77T/E and RC77N/E.

#	Ingenuity canonical pathways	−log⁡(*p* value)	Ratio	Molecules
1	Mitochondrial dysfunction	1.62*E* + 01	7.88*E* − 02	UCP2, RHOT2, NDUFS7, ATP5G1, ATP5F1, SOD2, ATP5L, NDUFB6, NDUFA8, UQCRQ, NDUFS5, NDUFV2, ATP5G3
2	Oxidative phosphorylation	1.33*E* + 01	9.62*E* − 02	ATP5L, NDUFB6, NDUFA8, UQCRQ, ATP5G1, NDUFS7, ATP5F1, NDUFS5, NDUFV2, ATP5G3
3	Superoxide radicals degradation	4.07*E* + 00	3.33*E* − 01	SOD2, SOD1
4	Amyotrophic lateral sclerosis signaling	4.07*E* + 00	4.12*E* − 02	SOD1, BCL2L1, BID, NEFL
5	Apoptosis signaling	2.91*E* + 00	3.41*E* − 02	BCL2L1, BID, BAK1
6	Molecular mechanisms of cancer	2.78*E* + 00	1.39*E* − 02	RHOT2, CDKN2A, BCL2L1, BID, BAK1
7	Docosahexaenoic acid (DHA) signaling	2.40*E* + 00	5.13*E* − 02	BCL2L1, BID
8	Aldosterone signaling in epithelial cells	2.24*E* + 00	1.99*E* − 02	HSPA1A/HSPA1B, HSPD1, HSP90AA1
9	Myc mediated apoptosis signaling	2.07*E* + 00	3.45*E* − 02	CDKN2A, BID
10	PEDF signaling	1.90*E* + 00	2.82*E* − 02	SOD2, BCL2L1

**Table 3:** Top upstream regulators and target molecules in the regulatory network.

Upstream regulator	Molecule type	*p* value of overlap	Target molecules in dataset
RBM5	Other	4.25*E* − 08	ATP5G3, CDKN2A, HSP90AA1, NDUFV2, UCP2
AIFM1	Enzyme	1.43*E* − 05	NDUFB6, NDUFS7
DEFB103A/DEFB103B	Other	1.43*E* − 05	BCL2L1, BID
BTG2	Transcription regulator	1.32*E* − 04	SOD1, SOD2
SHC1	Kinase	3.10*E* − 04	BAK1, BCL2L1
NFE2L2	Transcription regulator	4.92*E* − 04	SOD1, SOD2
APP	Other	7.13*E* − 04	DNM1L, HSPA1A/HSPA1B
RelB	Transcription regulator	8.83*E* − 04	BCL2L1, SOD2
HSF1	Transcription regulator	1.21*E* − 03	BCL2L1, HSP90AA1, HSPA1A/HSPA1B
SOCS1	Other	1.99*E* − 03	BAK1, BCL2L1
Igm	Complex	2.13*E* − 03	BAK1, CDKN2A
IGFBP7	Transporter	2.21*E* − 03	SOD2
NCF2	Enzyme	2.21*E* − 03	SOD2
mir-491	MicroRNA	2.21*E* − 03	BCL2L1
SIRPA	Phosphatase	2.21*E* − 03	BCL2L1
TERT	Enzyme	2.27*E* − 03	CDKN2A, SOD2
FAS	Transmembrane receptor	2.71*E* − 03	BID, SOD2
PPARA	Ligand-dependent nuclear receptor	2.86*E* − 03	CDKN2A, CPT2
IFNG	Cytokine	4.32*E* − 03	BAK1, BCL2L1, HSPA1A/HSPA1B, SOD2
LIN9	Other	4.41*E* − 03	CDKN2A
CASP3	Peptidase	4.41*E* − 03	BCL2L1
CUL4A	Other	4.41*E* − 03	SOD2
BTG3	Other	4.41*E* − 03	CDKN2A
NOXO1	Other	4.41*E* − 03	SOD2
CBX8	Other	4.41*E* − 03	CDKN2A
RAC2	Enzyme	4.41*E* − 03	BCL2L1
PRKACA	Kinase	4.41*E* − 03	CDKN2A
NEU2	Phosphatase	4.41*E* − 03	BCL2L1
S100A11	Kinase	4.41*E* − 03	HSPA1A/HSPA1B
BACH2	Enzyme	4.41*E* − 03	BCL2L1
CYBA	Other	4.41*E* − 03	CDKN2A
CFLAR	Transcription regulator	4.41*E* − 03	CDKN2A
NEU2	Enzyme	4.41*E* − 03	SOD2

**Table 4:** Patient clinical and pathological characteristics: CGG, clinical grade Gleason score; PGGS, pathological grade Gleason score.

#	Age	CGG1/2	CGGS	PathStageT	PGG1/2	PGGS	PrePSA
1	64	4 + 3	7	pT3b	4 + 5	9	9.8
2	62	3 + 4	7	pT2c	3 + 3	6	5.80
3	55	4 + 5	9	pT3b	4 + 5	9	6.750
4	49	4 + 3	7	pT3b	4 + 5	9	8.0
5	52	3 + 4	7	pT3b	3 + 3	6	5.4

## Abbreviations

cDNA: Complementary deoxyribonucleic acid
IPA: Ingenuity Pathway Analysis
qRT-PCR: Quantitative real-time polymerase chain reaction
RNA: Ribonucleic acid
ROS: Reactive oxygen species
SOD2: Superoxide dismutase 2
UCP2: Uncoupling protein 2.
