# Supplementary material for: Mitochondria Biogenesis and Bioenergetics Gene Profiles in Isogenic Prostate Cells with Different Malignant Phenotypes
Source: Biomed Res Int. 2016 Jul 10;2016:1785201. doi: 10.1155/2016/1785201 (PMC4958422; doi:10.1155/2016/1785201)

Supplemental Figure Legends

Supplemental Figure 1. RT-PCR showing the ΔΔCt values for NEFL in RC77N/E and RC77T/E cells. There is a significant down regulation of NEFL gene in the malignant RC77T/E cells compared to the non-malignant RC77N/E cells.

Supplemental Figure 2. The NEFL gene copy numbers in human prostate tissue samples in The Cancer Genome Atlas (TCGA). The copy numbers of the gene are significantly lower in a majority of the cases from both acinar prostate adenocarcinoma and prostate adenocarcinoma cases.

Supplemental Figure 3. Regulatory network of genes by IPA. The network of the genes was derived from the forty-seven differentially transcribed genes. The genes that are depicted in color present the differentially transcribed genes whereas the genes depicted in white were no change in transcribed genes. Red and shades of red represent upregulated genes whereas green represent down regulated genes. Full lines imply a direct action between two nodes, while the dotted lines illustrate an indirect relationship between two nodes. Blue lines represent inhibition whereas mauve lines represent activation.

Supplemental Figure 1.

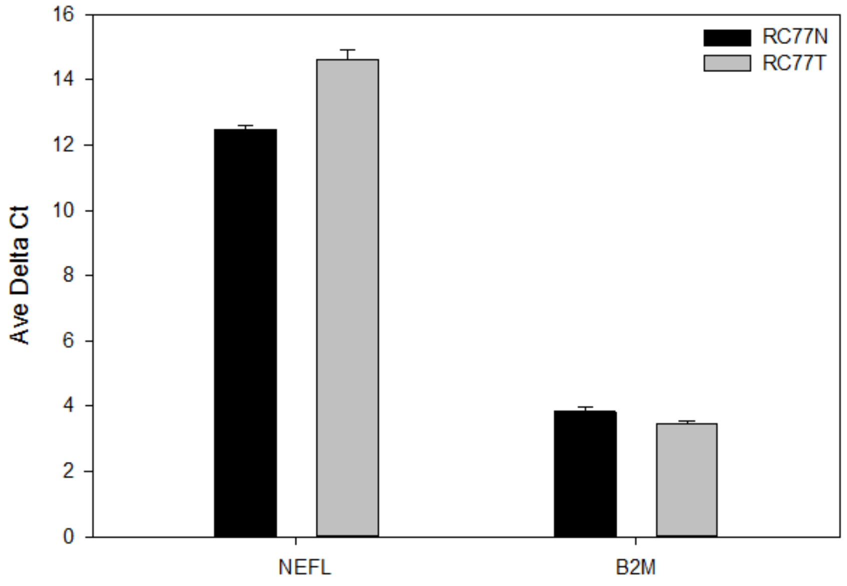

Supplemental Figure 2.

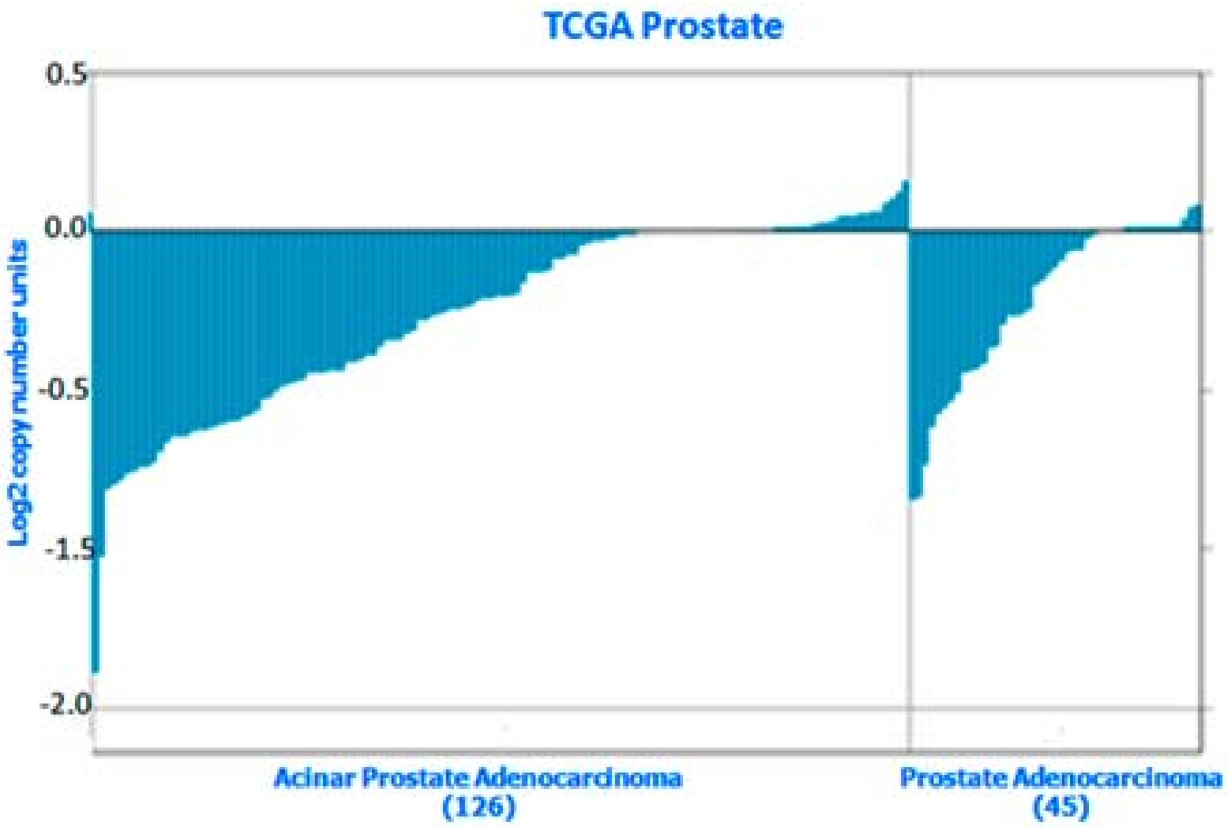

Supplemental Figure 3.

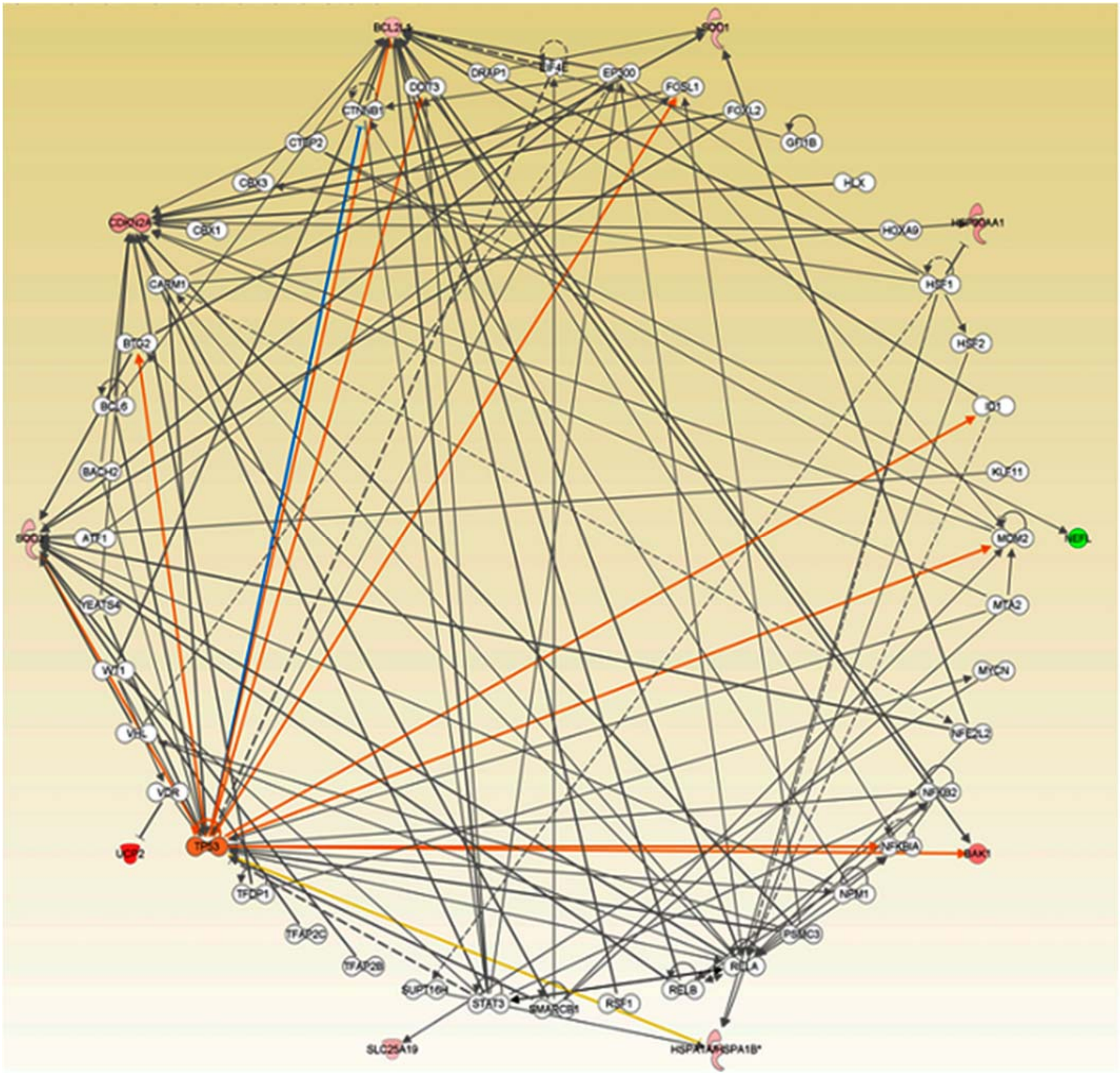

Supplement: Supplementary file 1 — RTPCR was performed for the 168 genes in the arrays and the results were analyzed by analyzed by the ΔΔCt method. Supplemental Figure 1 shows the ΔΔCt values for NEFL which is differentially expressed between the two cell lines. There is a significant down regulation of NEFL gene in the malignant RC77T/E cells compared to the non-malignant RC77N/E cells. The Cancer Genome Atlas (TCGA) was queried to determine NEFL gene copy numbers in human prostate tissue samples. The copy numbers of the gene are significantly lower in a majority of the cases from both acinar prostate adenocarcinoma and prostate adenocarcinoma cases (Supplemental Figure 2). Ingenuity pathway analysis was used to generate regulatory networks of the differentially transcribed genes between the two cell lines and the networks are summarized in Supplemental Figure 3. [file 1785201.f1.pdf]
